# Multi-Epitope Vaccine Design against Monkeypox Virus via Reverse Vaccinology Method Exploiting Immunoinformatic and Bioinformatic Approaches

**DOI:** 10.3390/vaccines10122010

**Published:** 2022-11-25

**Authors:** Kunal Bhattacharya, Israa M. Shamkh, Mohammad Shahbaz Khan, Marwa M. Lotfy, Jean Bosco Nzeyimana, Reem Fawaz Abutayeh, Nadia M. Hamdy, Dalia Hamza, Nongmaithem Randhoni Chanu, Pukar Khanal, Atanu Bhattacharjee, Emad B. Basalious

**Affiliations:** 1 Pratiksha Institute of Pharmaceutical Sciences, Guwahati 781026, Assam, India; 2 Royal School of Pharmacy, The Assam Royal Global University, Guwahati 781035, Assam, India; 3 Botany and Microbiology Department, Faculty of Science, Cairo University, Giza 12613, Egypt; 4 Chemo and Bioinformatics Lab, Bio Search Research Institution, BSRI, Giza 12613, Egypt; 5 Children’s National Hospital, Washington, DC 20010, USA; 6 Faculty of Pharmacy, Zagazig University, Zagazig 44511, Egypt; 7 College of Animal Science and Technology, Anhui Agricultural University, Hefei 230036, China; 8 Department of Pharmaceutical Chemistry and Pharmacognosy, Applied Science Private University, Amman 11931, Jordan; 9 Biochemistry Department, Faculty of Pharmacy, Ain Shams University, Abassia, Cairo 11566, Egypt; 10 Zoonoses Department, Faculty of Veterinary Medicine, Cairo University, Giza 12211, Egypt; 11 Faculty of Pharmaceutical Science, Assam Downtown University, Guwahati 781026, Assam, India; 12 Department of Pharmacology, NGSM Institute of Pharmaceutical Sciences, NITTE Deemed-to-be University, Mangalore 575018, Karnataka, India; 13 Department of Pharmaceutics and Industrial Pharmacy, Faculty of Pharmacy, Cairo University, Al Kasr El-Aini, Cairo 11562, Egypt

**Keywords:** monkeypox virus, multi-epitope vaccine, reverse vaccinology, immunoinformatics, bioinformatics

## Abstract

(1) Background: The monkeypox virus is a zoonotic orthopox DNA virus that is closely linked to the virus. In light of the growing concern about this virus, the current research set out to use bioinformatics and immunoinformatics to develop a potential vaccine against the virus. (2) Methods: A multiepitope vaccine was constructed from the B-cell and T-cell epitopes of the MPXVgp181 strain using adjuvant and different linkers. The constructed vaccine was predicted for antigenicity, allergenicity, toxicity, and population coverage. In silico immune simulation studies were also carried out. Expression analysis and cloning of the constructed vaccine was carried out in the pET-28a(+) vector using snapgene. (3) Results: The constructed vaccine was predicted to be antigenic, non-allergenic, and non-toxic. It was predicted to have excellent global population coverage and produced satisfactory immune response. The in silico expression and cloning studies were successful in *E. coli*, which makes the vaccine construct suitable for mass production in the pharmaceutical industry. (4) Conclusion: The constructed vaccine is based on the B-cell and T-cell epitopes obtained from the MPXVgp181 strain. This research can be useful in developing a vaccine to combat the monkeypox virus globally after performing in-depth in vitro and in vivo studies.

## 1. Introduction

In the democratic republic of Congo, a DNA virus known as monkeypox virus was found to be infecting humans in 1970 [1]. Despite being endemic, research and development regarding this virus were neglected for a long time. Recently, since May 2022, several infections in humans related to the monkeypox virus have been reported around the globe, which has made this virus an emerging threat to the human population. Monkeypox virus was classified as an “evolving threat of moderate public health concern” [2,3]. The monkeypox virus can spread from person to person through close communication with skin lesions, large respiratory droplets, and probably also through contaminated fomites [4]. There is no convincing evidence that the virus is passed from a sexual partner through seminal or vaginal fluids. There have been reports of fetal deaths as well as vertical transmission of the virus [5]. In the majority of instances, fever is the initial sign of sickness. This is often followed by the emergence of many papulopustular, vesiculopustular, and ulcerative lesions over the face and body, in addition to severe lymphadenopathy [6,7]. Pneumonitis, encephalitis, keratitis, and secondary bacterial infections are all examples of complications that can arise. There are presently no authorized therapies available for human monkeypox; however, two medications, brincidofovir and tecovirimat, both of which are orally bioavailable, were approved by the USFDA for the management of smallpox in preparation for a potential act of bioterrorism [8,9,10]. Neither drug has been tested for its effectiveness in human clinical trials; however, animal studies involving both drugs have shown that they are effective against other orthopoxviruses. Patients diagnosed with monkeypox in the United Kingdom are cared for in facilities that have been designated as HCID (high consequence infectious disease) treatment centers. These facilities are part of a national network that is responsible for coordinating care for patients with HCIDs [11]. Vaccine production using traditional methods is both time-consuming and relatively expensive [12]. For developing a novel vaccine construct for the monkeypox virus, computational studies-based vaccine design can be used by researchers and scientists to experimentally test against the monkeypox pathogen [13,14,15,16]. The multi-epitope vaccine design works with genetic information and makes use of a series of filters that are based on experimental data in order to select the antigenic epitopes that are most suitable for testing in experimental settings [17,18,19,20]. In this specific investigation, subtractive proteomics and reverse vaccinology methods were applied with the purpose of locating vaccine antigenic epitopes that were viable for application in the construction of a chimeric vaccine. These epitopes were ultimately put to use in the vaccine. As a result, we have reason to expect that the findings of this study could be of value to experimentalists in the process of developing an effective vaccine against the monkeypox virus.

## 2. Materials and Methods

### 2.1. Physiochemical Characteristic Assessment of Monkeypox Virus (MPXV) Vaccine Candidate

From the database maintained by the National Center for Biotechnology Information (NCBI), a protein dataset of MPXV was acquired (https://www.ncbi.nlm.nih.gov/protein/ (accessed on 18 June 2022)) and analyzed for prospective vaccine candidates. MPXVgp181 genome of monkeypox virus (accession number USJ88433.1) was chosen from the NCBI database because of the smaller number of transmembrane helices present in the structure, which was calculated using TMHMM 2.0 (https://services.healthtech.dtu.dk/service.php?TMHMM-2.0 (accessed on 18 June 2022)) [21]. Less transmembrane helices make it easier to clone and express proteins [22]. Furthermore, it shares no specific amino acid sequences with any protein discovered in the *Homo sapiens* genome, which was verified by subjecting the sequence to NCBI using NCBI BLASTP [23]. BioEdit, a sequence alignment editor, was used to examine the MPXVgp181 protein’s amino acid composition. [24]. ExPASy Protparam (https://web.expasy.org/protparam/ (accessed on 18 June 2022)) [25] was used to analyze the protein of interest in terms of its physicochemical properties. For secondary structure prediction, we used the PDBsum server (http://www.ebi.ac.uk/thornton-srv/databases/pdbsum/Generate.html (accessed on 19 June 2022)) [26]. Using VaxiJen 2.0 (http://www.ddg-pharmfac.net/vaxijen/ (accessed on 19 June 2022)) [27], we were able to identify the antigenic protein. The most antigenic protein was selected for subsequent studies. Evaluation of the samples for allergenicity was performed using the AllerTOP v2.0 (https://www.ddg-pharmfac.net/AllerTOP/ (accessed on 19 June 2022)) web server [28]. The protein’s tertiary structure was predicted using I-TASSER (https://zhanggroup.org/I-TASSER/ (accessed on 20 June 2022)) (Iterative Threading ASSEmbly Refinement), and the highest C-score model was deemed the most reliable [29].

### 2.2. B-Cell Epitope Mapping

B-cell epitopes were predicted using the IEDB linear epitope prediction tool (v2.0) with the default settings (https://services.healthtech.dtu.dk/service.php?BepiPred-2.0 (accessed on 23 June 2022)). This program employs a complex algorithm derived from the three-dimensional protein structures of antigen-antibody complexes [30]. This technique relies solely on epitope data derived from crystalline structures, and it is widely held that it is superior to alternatives in terms of quality, accuracy, and power [30]. The IEDB’s Bepipred 2.0 web server was used to conduct the analysis.

### 2.3. T-Cell Epitope Mapping

Predictions of MHC-I epitopes were made using the IEDB MHC-I binding predictions tool (http://tools.iedb.org/mhci (accessed on 23 June 2022)) [31]. Sequences were submitted in FASTA format, and the ANN 4.0 method was chosen as the prediction method. Humans were identified as the host species. All other options and parameters were left at their default settings, and XHTML tables were selected as the output format. Additionally, the MHC-II epitopes were predicted using the IEDB MHC-II binding predictions tool (http://tools.iedb.org/mhcii (accessed on 23 June 2022)) [32]. The FASTA sequence was uploaded, and NN-align 2.3 was selected as the prediction method. The HLA-DR was chosen as a species/locus pair, and all of the alleles were selected using the default length values for each species/locus. Other parameters were kept at their defaults, and XHTML table was chosen as the output format.

### 2.4. Analysis of Selected T- and B-Cell Epitopes for Antigenicity, Allergenicity, and Toxicity

At a threshold of 0.4, the VaxiJen 2.0 server (http://www.ddg-pharmfac.net/vaxijen (accessed on 24 June 2022)) was utilized to make a prediction about the antigenicity of T-cell and B-cell epitopes based on the physicochemical features of proteins [27]. In addition, the allergenicity of T- and B-cell epitopes were evaluated with the help of a program called AllerTOP (https://www.ddg-pharmfac.net/AllerTOP (accessed on 24 June 2022)) [28]. Furthermore, toxicity testing was performed with the use of the ToxinPred2 server (https://webs.iiitd.edu.in/raghava/toxinpred2/ (accessed on 24 June 2022)) [33].

### 2.5. Population Coverage Analysis

Using the population coverage analysis tool available on the IEDB website, we analyzed the population coverage of the selected epitopes (http://tools.iedb.org/population (accessed on 26 June 2022)) [34]. Selected MHC-I and MHC-II epitope data in combined form were uploaded, and the selection process has taken into account for all regions of the world.

### 2.6. Vaccine Construction

To create the multi-epitope vaccine, the adjuvant sequence was obtained from the UniProt database (https://www.uniprot.org/ (accessed on 27 June 2022)), and then all of the available epitopes were used to produce the vaccine. The adjuvant 50S ribosomal protein L7/L12 (UniProt ID: P9WHE3) was utilized to attach to the B-cell epitopes by the use of the EAAAK linker, which was then coupled to the T-cell epitopes containing the cytotoxic T-lymphocytes and helper T-lymphocytes through the use of the KK, AAY, and GPGPG linkers [35].

### 2.7. Antigenicity, Allergenicity, and Toxicity Profiling of Constructed Vaccine

VaxiJen 2.0 (http://www.ddg-pharmfac.net/vaxijen (accessed on 27 June 2022)) was used to analyze the vaccine design for antigenicity [27], whereas AllerTOP was used to predict the allergenicity of the multi-epitope vaccine. (https://www.ddg-pharmfac.net/AllerTOP (accessed on 27 June 2022)) [28]. ToxinPred (https://webs.iiitd.edu.in/raghava/txnpred/ (accessed on 27 June 2022)) [33] was also used to evaluate the toxicity profile.

### 2.8. Solubility and Physicochemical Characteristics Analysis of Vaccine Construct

Prediction of the propensity of the constructed vaccine protein’s solubility upon overexpression in *E. coli* was performed using SOLpro [36] (http://scratch.proteomics.ics.uci.edu/ (accessed on 28 June 2022)), and ExPASy Protparam (https://web.expasy.org/protparam/protparam-doc.html (accessed on 28 June 2022)) was used to view the physicochemical properties of the constructed vaccine [25].

### 2.9. Secondary and Tertiary Structure Extrapolation; Validation and Refinement of the Derived Tertiary Structure of Vaccine Construct

The secondary and tertiary structures of the multi-epitope vaccine were extrapolated with the help of PDBsum (http://www.ebi.ac.uk/thornton-srv/databases/pdbsum/Generate.html (accessed on 28 June 2022)) and I-TASSER (https://zhanggroup.org/I-TASSER/ (accessed on 28 June 2022)) [26,29] to get an idea of the primary helices, sheets, strands, beta turns, gamma turns, and disulfide bonds in the protein structure. GalaxyRefine, a web server, is one of the most reliable resources for refining the tertiary structures. As part of the refinement method, initially, side chain rebuilding and repacking were performed. Validation and refinement of the vaccine protein’s tertiary structure were performed using the GalaxyRefine server (https://galaxy.seoklab.org/ (accessed on 29 June 2022)) [37].

### 2.10. Validation and Molecular Docking of Vaccine’s 3D Structure

MolProbity (http://molprobity.biochem.duke.edu/ (accessed on 4 July 2022)) was utilized in order to validate the quality of the 3D structure [38]. The online docking server Cluspro2.0 (https://cluspro.bu.edu/ (accessed on 5 July 2022)) was used to carry out the docking study between the ligand-binding domain of the TLR2 receptor (PDB: 2Z7X) and the designed vaccine construct. This study was carried out in order to determine whether or not the designed vaccine construct would be effective. Because of the wide variety of specialized search options that could be adapted to fulfill the user’s particular requirements, Cluspro2.0 was considered one of the most effective docking servers [39].

### 2.11. Molecular Dynamics Simulations

Docked protein and ligand–protein complexes were subjected to MD simulations using Schrödinger, L.L.C.’s Desmond 2020.1. With period boundary salvation box dimensions of 10 Å × 10 Å × 10 Å, SPC water molecules and the OPLS-2005 force field were utilized in this system. The physiological environment was mimicked by adding 0.15 M of NaCl solution and Na^+^ ions to the system to neutralize the charge. Retraining with the protein–protein complex began with a 10-ns NVT ensemble equilibration to ensure system stability. The previous phase was followed by a rapid equilibration and reduction using a 12-ns NPT ensemble run. The Nose–Hoover chain coupling technique [40,41] was used to build up the NPT ensemble, and the experiment was conducted at 37 degrees Celsius for 1.0 picoseconds at a pressure of 1 bar [42]. In this study, a 2 fs time step was used. The Martyna–Tuckerman–Klein barostat technique was used to regulate pressure using a relaxation time constant of 100 fs. Long-range electrostatic interactions were calculated using Ewald’s particle mesh method, using a coulomb interaction radius of 9 nm. The RESPA integrator was used to compute the bonded forces for each trajectory, and a time step of 2 fs was used for individual trajectories. The whole production run lasted for one hundred nanoseconds. Using metrics such as the root mean square deviation (RMSD), root mean square fluctuation (RMSF), radius of gyration, and hydrogen bonds number, calculations were carried out to monitor the stability of MD simulations.

### 2.12. Codon Optimization for Vaccine Peptide Expression Studies

Codon optimization was performed using the Java codon adaption tool (http://www.jcat.de/ (accessed on 10 July 2022)), an online web-based server [43], after the sequence was reverse-translated using the EMBOSS 6.0.1 (https://www.ebi.ac.uk/Tools/emboss/ (accessed on 10 July 2022)) backtranseq program [44]. These steps were taken because *E. coli* uses a different set of codons than the native host, making it difficult to produce the synthesized peptide in this strain. This was achieved by copying and pasting the construct’s nucleotide sequence while making selections were made to eliminate rho-independent transcription termination, bacterial ribosome binding, and enzyme cleavage site restoration. Using the codon adaption index (CAI) score and the proportion of high GC-content codons, we evaluated the quality of the expression based on these criteria. It is generally agreed that a CAI score between 0.8 and 1.0 is favorable, with a value of 1.0 being ideal. Because values outside this range have a deleterious impact on transcriptional and translational activity, the optimal GC content range is between 30 and 60 percent. In this research, the *E. coli* pET-28a (+) expression vector was used. SnapGene (https://www.snapgene.com/ (accessed on 11 July 2022)) was used in silico PCR to amplify the construct.

### 2.13. Immune Simulation

Immune response induction utilizing the C-ImmSim server (http://kraken.iac.rm.cnr.it/C-IMMSIM/ (accessed on 12 July 2022)) validated the effectiveness of the vaccine [45]. In order to identify immunological epitopes and immune interactions, this server uses a position-specific scoring matrix (PSSM). All of the default options were utilized throughout the experiment.

## 3. Results

### 3.1. Antigenicity and Physiochemical Characteristic Evaluation of MPXV Virus Protein

**Figure 1:**
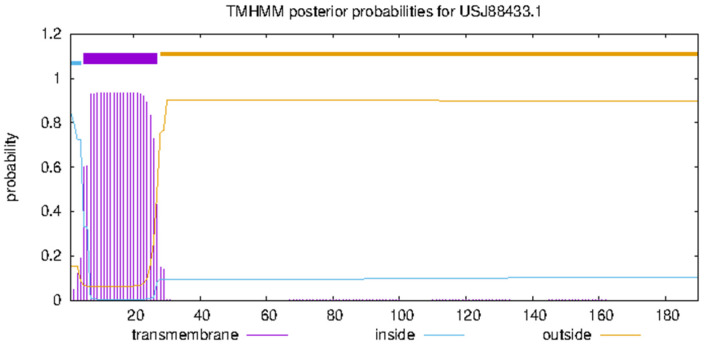
Transmembrane helices prediction in MPXVgp181 protein by TMHMM.

**Figure 2:**
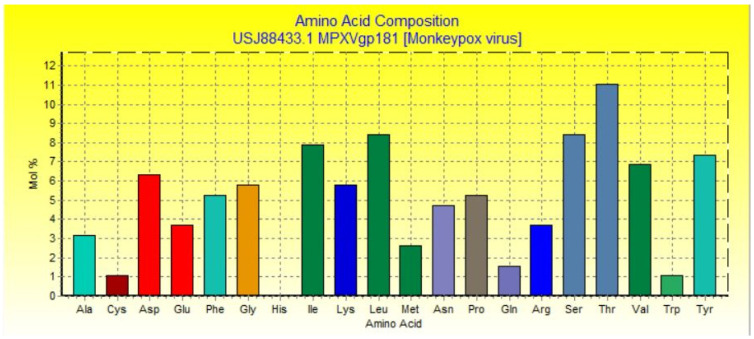
Amino acid composition of MPXVgp181 protein.

The sequence of the MPXVgp181 virus protein (NCBI accession no: USJ88433.1) was obtained with the purpose of creating a vaccine based on a smaller number of transmembrane helices present in the structure obtained using TMHMM 2.0 to facilitate the cloning of the vaccine construct in the vector. It was shown that the residues 28-190 do not belong to the transmembrane region. However, residues 1-4 were discovered deep inside the nucleoprotein’s core, whereas residues 5–27 were located within the transmembrane domain (Figure 1). Amino acid composition analysis provided by BioEdit showed the presence of 19 amino acids in which threonine had the maximum percentage (Figure 2).

**Figure 3:**
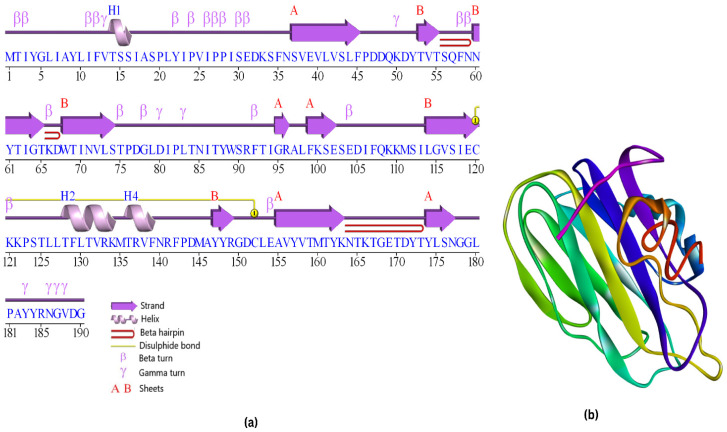
(**a**) Secondary structure prediction of MPXVgp181 strain protein predicted by PDBsum. (**b**) Tertiary structure of MPXVgp181 strain protein predicted by I-TASSER.

Using the VaxiJen 2.0 online server, we developed an antigenicity estimate for the viral protein. We selected a threshold of 0.5 to maximize accuracy. MPXVgp181 was predicted to be an antigen with an antigenic score of 0.6234. Physiochemical characteristics of MPXVgp181 were determined using ProtParam software. The investigation showed that it was composed of 190 different amino acids, and the predicted molecular weight was 21,546.67 Da. The theoretical isoelectric point (PI) was calculated as 5.48, indicating that the protein is charged evenly throughout at that pH at that value. Protparam determined the protein to be stable since its instability index (II) is 30.96. This protein has a high aliphatic index of 86.63, making it stable across a wide temperature range. The molecular formula is C_984_H_151_4N_236_O_292_S_7_, and it is used to determine the total number of atoms consisting of carbon, hydrogen, nitrogen, oxygen, and sulfur. The protein’s GRAVY score of −0.077, which is negative, suggests that it is hydrophobic in nature. It was projected that mammalian reticulocytes would have a half-life of around 30 h. Additionally, the MPXVgp181 protein was found to contain four helices, two sheets, ten strands, twenty beta turns, eight gamma turns, and one disulfide bond that was predicted for it by PDBsum and I-TASSER for its secondary and tertiary structures, respectively (Figure 3).

### 3.2. B-Cell Epitope Mapping

**Figure 4:**
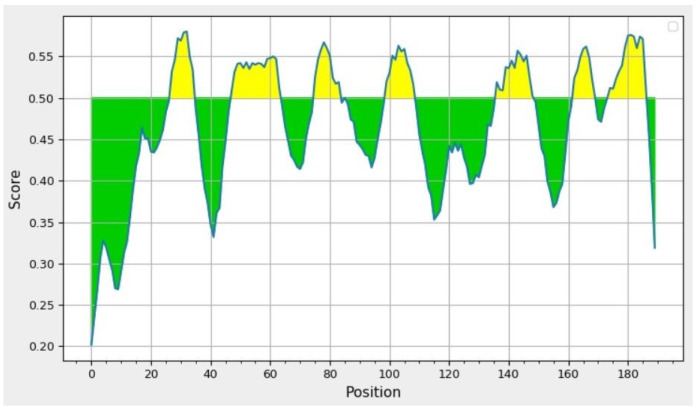
Bepipred linear epitope prediction of the MPXVgp181 protein.

B-cell epitopes have a substantial impact on the development of an immune response that is resistant to the infection caused by viruses. Potential B-cell epitopes contain changed features that educate B-cells to recognize and trigger a wide variety of immune responses to a specific viral infection. B-cells are able to recognize their targets and initiate immunological responses because of these properties. In particular, we planned to use methods that were based on amino acid screening for the exploration of potential B-cell epitopes in this work. Linear epitope prediction utilizing Bepipred and a threshold score of 0.500 predicted a total of eight linear epitopes in the yellow-coloured regions in Figure 4, of which four epitopes (LSNGGLPAYYRNG, KNTKTGE, PDGLDIPLT, and DQKDYTVTSQFNNYTIG) were finalized after discarding the remaining epitopes based on antigenicity and allergenicity analysis using the VaxiJen 2.0 and AllerTOP servers. The hidden Markov model-based technique is one of the more effective methods, and we used it to compute the linear epitopes. The minimum score for linear epitope prediction is 0.202, while the maximum score is 0.580. In addition to this, it was found that the average score for prediction was 0.467 (Figure 4, Table 1).

### 3.3. T-Cell Epitope Mapping

#### 3.3.1. Prediction of MHC Class-I Epitopes

We were able to study many different HLA alleles in humans by using Homo sapiens as our MHC source and the ANN 4.0 method. The output interface of this application is an HLA-binding affinity expressed in IC50nM units. A low IC50 value indicates that the epitope has a high affinity for the MHC Class-I alleles. It was shown that 152 of the identified epitopes had IC50 values < 100, indicating a significant propensity to interact with a wide variety of MHC Class-1 alleles. Of the total of 152 epitopes, 7 epitopes (AVYVTMTYK, FLTVRKMTRV, ITYWSRFTI, LTNITYWSR, VTMTYKNTK, YLIFVTSSI, and YVTMTYKNTK) were chosen because of their potential to interact with maximum MHC Class-1 alleles and antigenicity and allergenicity analysis (Table 2).

#### 3.3.2. Prediction of MHC Class-II epitopes

After investigating allergenicity, and antigenicity, a total of 4753 predicted epitopes were narrowed down to 8 epitopes (ITYWSRFTI, KMSILGVSI, LIFVTSSIA, LLTFLTVRK, LTFLTVRKM, SFNSVEVLV, YIPVIPPIS, and YVTMTYKNT) with IC50 values below 100 that were chosen for further research because of their positive and maximum number of interactions with MHC Class-II alleles (Table 3).

### 3.4. Vaccine Assembly

**Figure 5:**
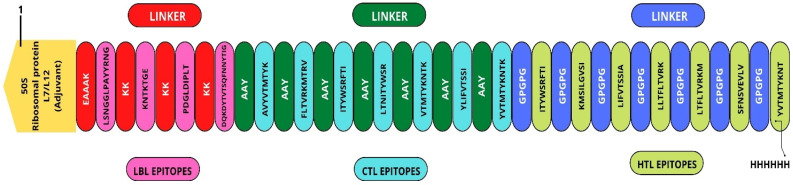
Multi-epitope vaccine construct.

A multi-epitopic vaccine chimera was created by employing a total of eighteen epitopes, four of which were B-cell epitopes, seven of which were MHC Class-I epitopes, and seven of which were MHC Class-II epitopes. During the construction process of the vaccine, the adjuvant known as 50S ribosomal protein L7/L12 (UniProt ID: P9WHE3) was used in order to trigger a targeted immunological response. An EAAAK linker was used to connect the primary B-cell epitope to the adjuvant. In addition, linkers of the KK type were used in order to connect B-cell epitopes, while linkers of the AAY type were utilized in order to connect MHC Class-I epitopes. GPGPG linkers were used in order to accomplish the goal of linking MHC Class-II epitopes. The sequence of the vaccine was altered by the inclusion of a 6x His tag at the very end of the vaccine construct to make the process of identifying and purifying the protein more straightforward. Figure 5 shows the amino acid sequence of the vaccine that was produced as a consequence. The vaccine has a molecular weight of 40403.33 Da and contains 377 amino acid residues. HHHHHH is the polyhistidine tag which can be used for enhanced expression in *E. coli*.

### 3.5. Population Coverage Analysis

**Figure 6:**
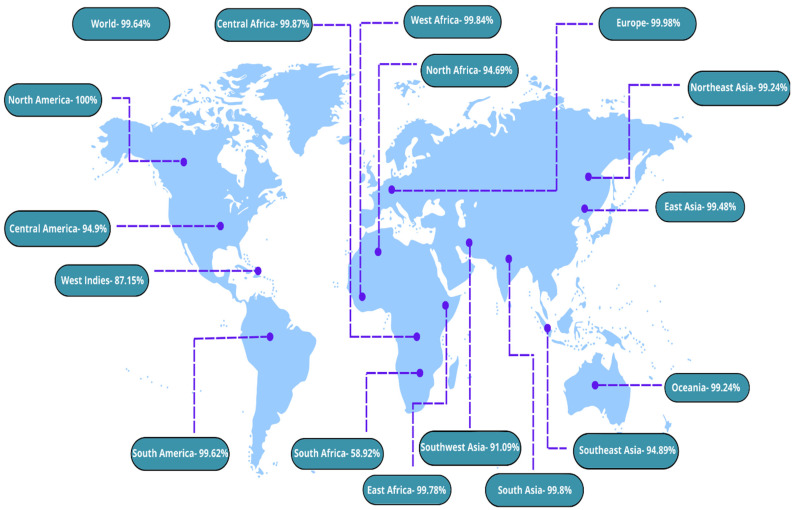
Population coverage analysis.

Using the IEDB population coverage analysis tool, it was possible to identify the most frequent candidate epitopes for each of the coverage methods. This was performed by analyzing IEDB database information. The distribution of MHC and HLA alleles varies in different geographic regions of the globe as a direct consequence of the effect of numerous environmental factors. It is essential to consider population coverage as part of the process in order to develop a potentially effective vaccine that can be administered. North America had the most population exposure to MHC Class-I- and Class-II-combined alleles, which was assessed as 100%, while South Africa had the lowest population coverage, which was calculated as 58.92%. Figure 6 shows a summary of the data in detail.

### 3.6. Evaluation of Vaccine Protein’s Antigenicity, Allergenicity, and Toxicity

According to the results of the computations carried out by the VaxiJen 2.0 web server, it was anticipated that the antigenicity of the vaccine protein, when used in conjunction with the adjuvant, would be 0.6002. It has been determined, on the basis of the data, that the structure of the vaccine is antigenic. According to the findings of AllerTOP version 2, the protein included in the vaccine was shown to be non-allergenic regardless of whether or not the adjuvant was present in the formulation. The toxicity of the protein was tested using ToxinPred, and the results showed that it was non-toxic.

### 3.7. Solubility and Physicochemical Property Analysis of Multi-Epitope Vaccine Subunit

The physicochemical properties of the constructed vaccine were predicted with the use of the ExPASYProtParam server, and the findings showed a number of characteristics that were connected to the type of protein in question. The molecular weight (MW) of the component that was used in the multi-epitope vaccination was 40,403.33 Da. According to the results of the computations, the pI of the protein was anticipated at 9.38. The instability index (II) came in at 16.75, which suggests that the vaccine construct is quite stable. If this value is more than 40, it suggests that the protein is unstable. The fact that this protein had an aliphatic index of 82.36 when it was examined indicated that it could withstand high temperatures without degrading. According to the results provided by the SOLpro server, the solubility rate of our vaccine design was satisfactory, and it was given a score of 0.8728.

### 3.8. Vaccine’s Secondary Structure Extrapolation

**Figure 7:**
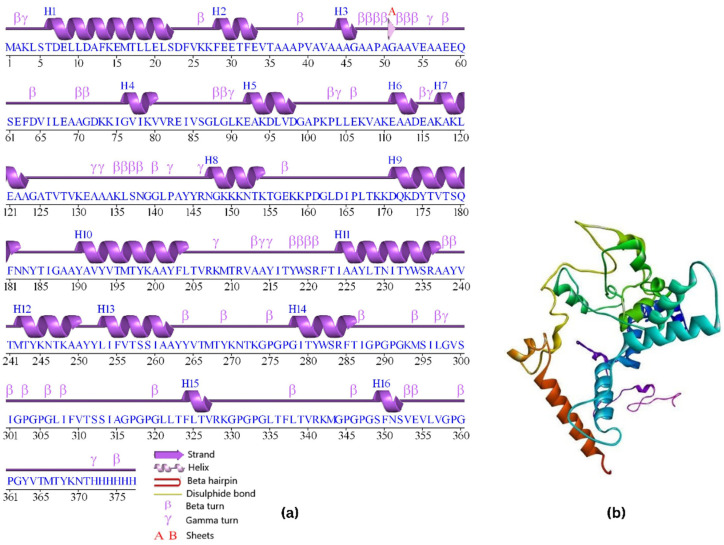
(**a**) Secondary structure of multi-epitope vaccine; (**b**) refined tertiary structure of multi-epitope vaccine.

Secondary structure extrapolation (Figure 7a) was accomplished through the use of the PDBsum server, which examined the protein’s actual nature and then extrapolated its secondary structure. Following analysis of the results, it was determined that it consists of 1 sheet, 2 strands, 16 helices, 49 beta turns, and 16 gamma turns.

### 3.9. Vaccine’s Tertiary Structure Extrapolation

In order to produce the most accurate model of the tertiary structure of the chimeric vaccine construct that was possibly attainable, the I-TASSER server was put to use. The models were predicted by using the top five threading templates, which were chosen on the basis of high coverage values so that the models could be more accurate. In this query, the model selected for further refinement was determined on the basis of the highest score in terms of coverage.

### 3.10. Tertiary Structure Refinement

Following the completion of the refining process, the Galaxy Refine tool generated a total of five different vaccine chimera models. Model 2 (Figure 7b) was chosen for further investigation because it looked the most promising out of the five models. During the process of refining, many characteristics were taken into account, such as GDT-HA (0.9595), RMSD (0.385), and MolProbity score (2.057). The Ramachandran score was anticipated at 90.4 percent, while the clash score was calculated as 10.0, and the score of poor rotamers was 0.7.

### 3.11. 3D Structure Validation

**Figure 8:**
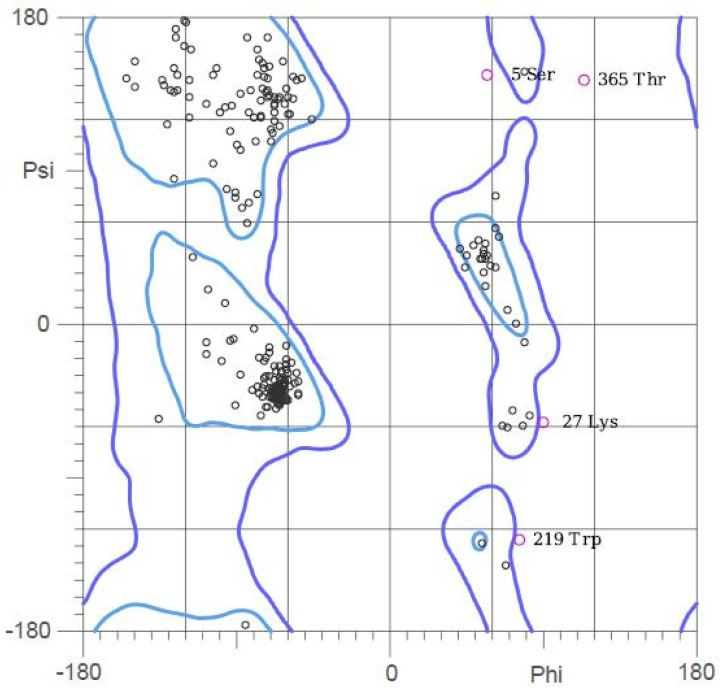
Ramachandran plot of refined multi-epitope vaccine.

The revised tertiary structure was checked using the MolProbity server. The protein’s structure was looked at, and a Ramachandran plot was made (Figure 8). Before refinement, 64.5% (242/375) of all residues were in the favored region, and 86.1% (323/375) of all residues were in the allowed region. After refinement was completed, the MolProbity results were better. Of all residues, 90.4% (339/375) were in the preferred region, 97.6% (366/375) were in the allowed region, 90.4% (339/375) were in favored (98%) regions, and 97.6% (366/375) were in allowed (>99.8%) regions.

### 3.12. Molecular Docking with TLR2

**Figure 9:**
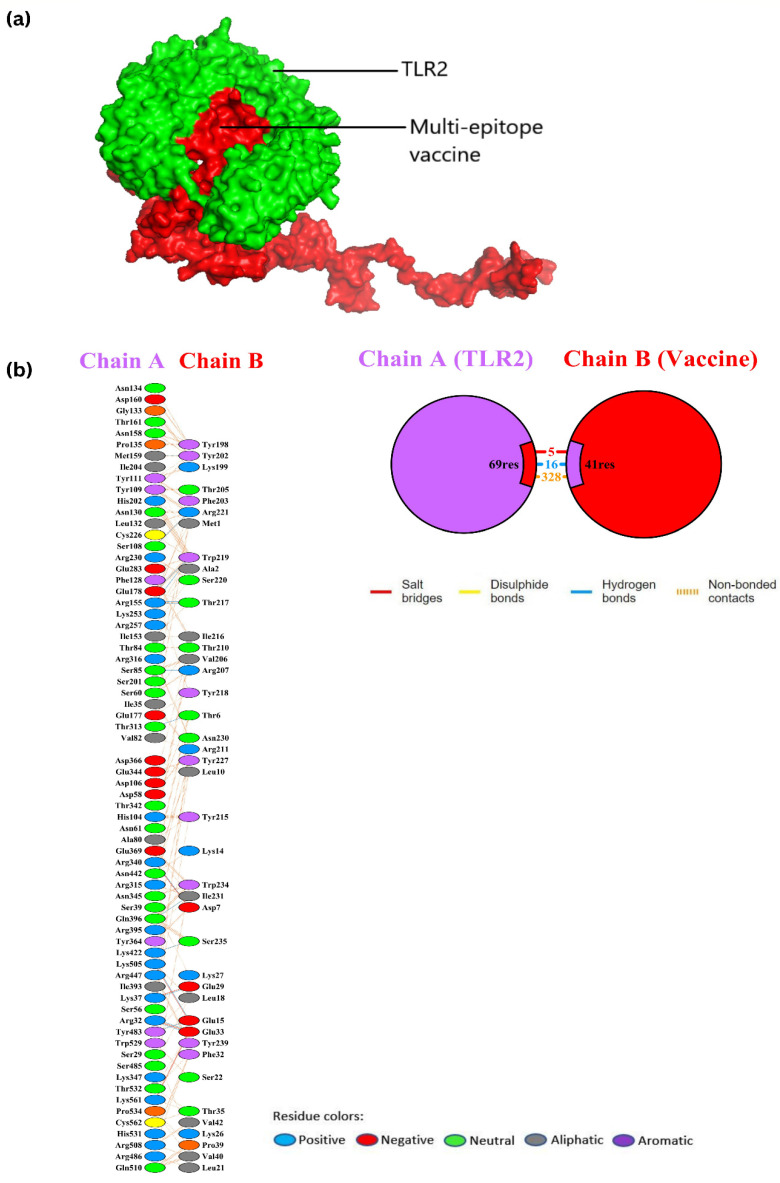
(**a**) Docked complex between TLR2 and multi-epitope vaccine; (**b**) protein–protein interaction of TLR2–vaccine complex.

Molecular docking was performed so that a prediction could be made of the interaction between the optimized vaccine construct and the ligand-binding domain of the immunological receptor TLR2. This was achieved by utilizing the online protein–protein docking server Cluspro2.0, which has been particularly created for protein–protein docking. Docking makes it possible to conduct parallel inspections of several models at the same time. It was decided to go with the protein–protein docked complex that had the maximum cluster size of 56, a center-weighted score of −917.4, and the lowest energy score of −1076.0. Between Chain A of TLR2 and Chain B, which represents the vaccine protein, the docked complex has 16 hydrogen bonds, 5 salt bridges, and 328 non-bonded contacts. Figure 9, Table 4 and Table 5 provide a summary of the findings on protein–protein interactions.

### 3.13. Molecular Dynamics Simulation

**Figure 10:**
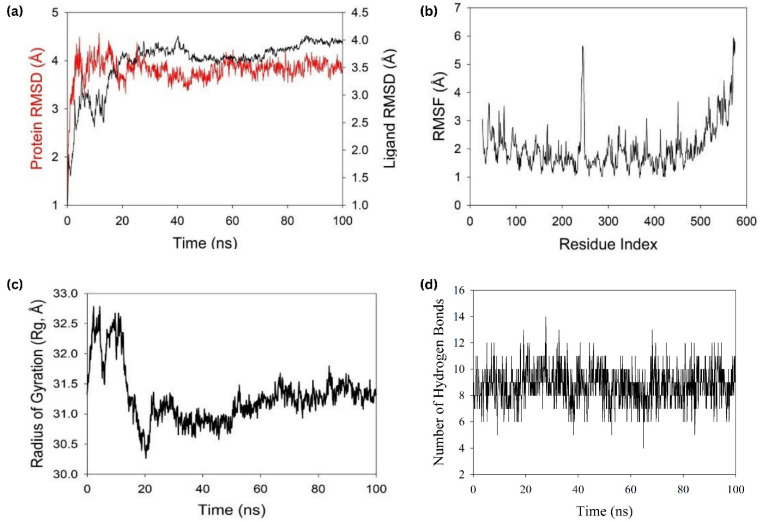
Analysis of MD simulation trajectories for 100 ns. (**a**) RMSD plot displaying. the molecular vibration of Cα backbone of protein (red) and ligand (black). (**b**) RMSF plot showing the fluctuations of respective amino acids throughout the simulation time 100 ns for protein. (**c**) Radius of gyration plots for the deduction of compactness of protein. (**d**) Number of hydrogen bonds formed between protein and ligand during 100 ns simulation time scale.

**Figure 11:**
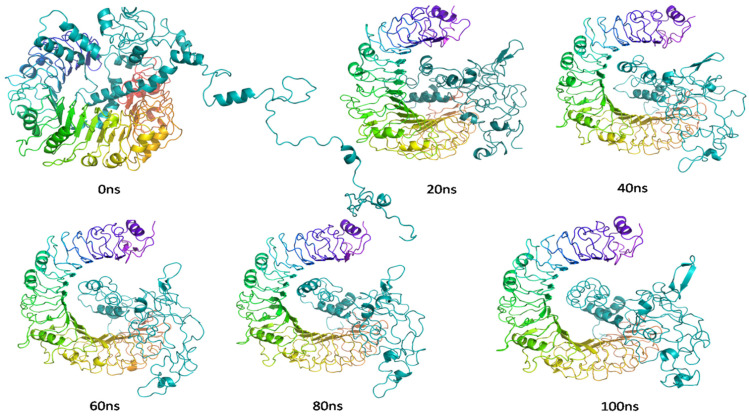
Snapshot of MD trajectories at different time interval for visualization of ligand–protein chain conformational changes into the binding cavity of protein chain.

Studies using molecular dynamics and simulation, also known as MD, were carried out with the goal of determining the stability and convergence of protein and ligand–protein interactions. When comparing the root mean square deviation (RMSD) measurements, the simulation lasting 100 ns showed a stable conformation. The root mean square deviation of the protein’s Cα-backbone showed an average variance of 3.8 Å. (Figure 10a) while the ligand–protein RMSD was observed to be 4 Å at the end of the simulation (Figure 10a). The overall RMS deviation was found to be within the acceptable range. During the simulation, RMSD graphs that remain stable are indicative of excellent convergence and stable conformations. As a result, it is possible to hypothesize that the protein and ligand–protein complex is highly stable as a result of the increased affinity between the two components. The plot for root mean square fluctuation (RMSF) showed that there were small spikes of fluctuation in the Cα-atoms of protein chains, with the exception of residues 240–260. These spikes could be due to the higher flexibility of the residues conformed into the loop region, whereas the rest of the residues fluctuated less throughout the entire 100 ns simulation upon binding with the ligand–protein (Figure 10b), indicating stable amino acid conformations during the simulation. The secondary structure of those fluctuating regions was found to be mostly loops and turns. These RMSF values are all within the range of what is considered acceptable. The compactness of the protein is measured using a metric called the radius of gyration. Cα -atoms in proteins showed a decrease in their radius of gyration (Rg), which went from 31.5 to 31.2 Å in this simulation study (Figure 10c). A very compact orientation of the protein in its ligand-bound form is indicated by a significant reduction in the peaks and a steady gyration (Rg) value. The substantial contact and stability of the complex are shown by the high number of hydrogen bonds that exist between the ligand and protein. The number of hydrogen bonds corroborated with docking studies. The average number of hydrogen bonds in the MD simulation was 12, whereas in docking 14 hydrogen bonds were similarly displayed (Figure 10d, Table 6). Specific numbers of salt bridges were also monitored and found Lys14 ligand–proteins with Asp58 with protein amino acid residue on a couple of occasions (Table 6). From the MD simulation trajectory, the binding energies of every 20 ns were determined using MMGBSA. The snapshots of every 20 ns are displayed in Figure 11. Free energy of binding of the ligand–protein with the protein at the beginning (0 ns) was found to be −30.33 kcal/mol. Here, the GLA domain can be seen bound at the pocket of A and B chains superficially (Figure 11), while at 20 ns, the domain seemed to move a bit into the binding cavity and bound with high affinity, and free energy binding was measured at −31.527 kcal/mol (Figure 11). The 40 ns and 60 ns structures of the ligand–protein displayed the movement deeper into the binding core of the protein as compared to the previous time (Figure 11). The free energies were calculated as −32.397 and −33.235 kcal/mol, respectively. At last, the highest binding affinity was achieved at 100 ns, where the entire domain moved inside the binding core and oriented for the highest stability (Figure 11). The free energy of binding was measured at −34.859 kcal/mol, with high affinity and more negative binding energies signifying higher stability of the complex.

### 3.14. Codon Optimization of Proposed Vaccine Peptide for Expression Analysis

**Figure 12:**
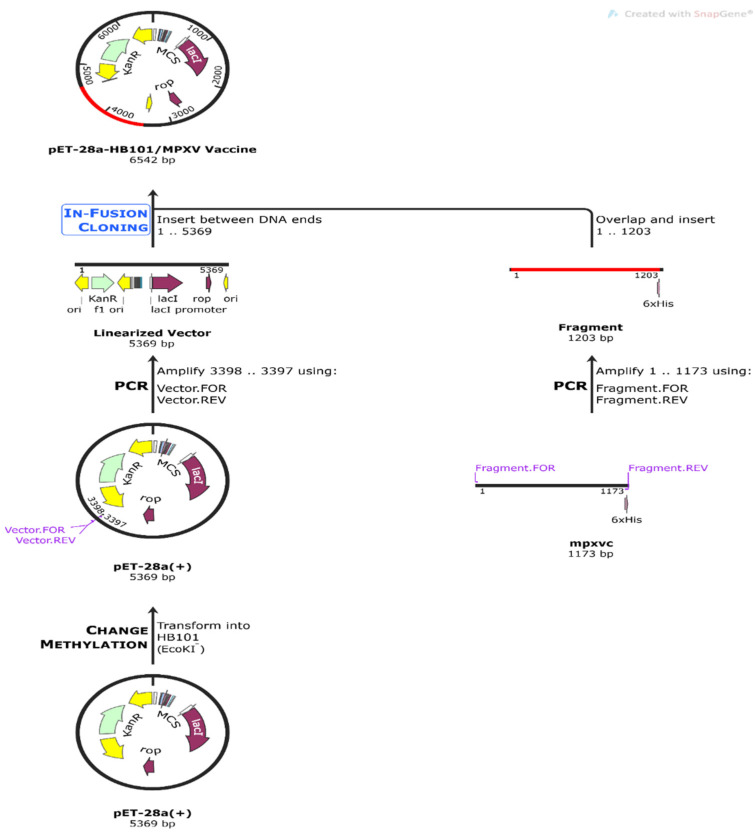
In silico PCR amplification and cloning of vaccine construct.

The Java codon adaptation tool, or JCat, was used to optimize the codons for maximal protein expression. With an estimated GC content of 50.72 percent, the optimized codon had a CAI of 0.98. These results, together with the fact that the GC concentration is between 30% and 60%, are indicative of stable vector expression in *E. coli*. The modified sequence was amplified using in silico PCR with the help of SnapGene and then cloned into a pET-28a(+) vector to create a recombinant plasmid (Figure 12).

### 3.15. Immune Simulation

**Figure 13:**
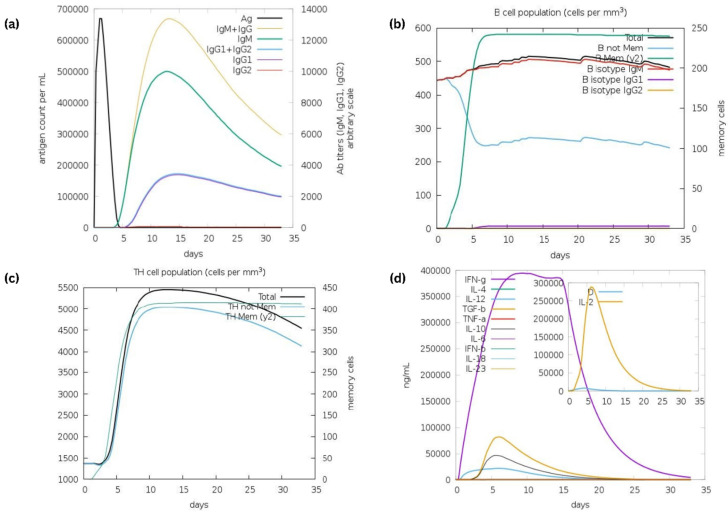
Immune simulation of multi-epitope vaccine: (**a**) immunoglobulin production on subsequent injection of antigens (shown by black lines); colored lines indicative of immune cells class; (**b**) shows changes in B-cell population and memory formation; (**c**) production of helper T-cells; (**d**) elevated rates of cytokines and interleukins for effective immune response.

For the purpose of carrying out the immune simulation, the C-ImmSim server was used. This demonstrates an immunological response that is comparable to a true immune response. A rise in levels of IgM+IgG was characteristic of the first reaction, which was then followed by increases in levels of IgM and IgG1+IgG2, respectively (Figure 13a). Both the secondary and tertiary stages of the immune response were distinguished by the presence of a significant number of B-cells (Figure 13b). In addition to this, the findings demonstrated the formation of memory cells after further exposure. Additionally, there was an increase in the number of helper (TH) cells (as seen in Figure 13c) as well as cytokines (Figure 13d).

## 4. Discussion

It is difficult to prevent an outbreak of MPXV, as evidenced by a documented rise in the number of cases of human MPXV and occasional clusters all over the globe. The currently available vaccines only provide a moderate level of protection against MPXV, particularly in younger children and individuals who already have a preexisting medical condition [1]. As a result, innovative treatment approaches are necessary for newly discovered MPXV infections. The creation of vaccines has benefited from developments in reverse vaccinology, as well as from the availability of genetic and proteomic data. In addition, the use of cutting-edge bioinformatics tools is more advantageous than the use of conventional research methods [46]. When it comes to designing and developing effective vaccines, epitope-based vaccines provide a novel treatment method thanks to their superior safety, efficacy, and logistical feasibility. There is a long history of success with vaccines that contain live or attenuated viruses, but they are linked to a variety of side-effects, such as autoimmune and allergic reactions. This has resulted in the use of immunoinformatics techniques as a means of eradicating such biosafety concerns, in addition to making use of time and cost savings opportunities. Peptide-based vaccine design has shown to be effective against a variety of viruses, including the Dengue virus, Chikungunya virus, Rhinovirus, and SLE virus, to name just a few others [47]. Multi-epitope vaccines may stimulate protective immune responses by targeting many conserved epitopes present in whole antigenic sequences. This allows them to avoid responses against unfavorable epitopes, which could potentially induce immunopathogenic or immune-modulating responses against the host [48,49]. There is currently no specific therapy for MPXV, and immunization against MPXV infection is the sole preventative intervention available. Using immunoinformatic approaches, the purpose of this work was to build an innovative multi-epitope MPXV vaccine that is capable of generating immunogenic responses in persons who are infected with the virus. Protein encoding for the MPXVgp181 (USJ88433.1) information was retrieved based on criteria such as antigenicity, non-allergenicity, and non-toxicity in order to locate T-cells and B-cells. This approach assesses vaccine candidates’ eligibility for experimental validation [50]. Since the PDB structure of the MPXVgp181 protein was not available in any of the protein data banks, the structure of the protein had to be constructed with the use of a bioinformatics tool, i.e., I-TASSER. An efficient multi-epitope vaccine should be developed with the intention of including epitopes that are able to produce CTL, HTL, and B-cell epitopes as well as induce effective responses to a particular virus [51]. We have incorporated B-cell epitopes because of their role in antibody production [52]. Although antigens can eventually overcome the humoral response from memory B-cells over time, but T-cell immunity, or cell-mediated immunity, typically results in extended protection [53]. Cytotoxic T-lymphocytes confine pathogens spread by the identification of virus and elimination of infected cells by releasing specialized cytokines that fight viruses [54]. Therefore, the vaccine’s B- and T-cell epitopes were anticipated in the multi-epitope vaccine construct. In order to develop a vaccine construct, B-cell epitopes, MHC-I epitopes, and MHC-II epitopes were chosen and then connected together utilizing a variety of linkers and adjuvant peptide sequences derived from 50S ribosomal protein L7/L12 (UniProt ID: P9WHE3). The suggested multi-epitope construct received high antigenicity scores when tested with VaxiJen version 2.0. The vaccine construct that was designed was non-allergenic. Because of its immunological features, it has greater potential to become a vaccine candidate. ExPASy Protparam and SOLpro were used to evaluate the physicochemical properties of the projected vaccine construct, and the results indicated that it has a high degree of stability and solubility. Finding information on the structure of the virus by investigating the ways in which antigens and receptor molecules interact with one another is essential for the development of vaccines. I-TASSER was used to make a prediction of the vaccine construct’s 3D structures, and then the GalaxyRefine server was used to refine the structure. The improved three-dimensional structural analysis demonstrated that the intended structure is structurally stable. The refined vaccine construct demonstrated the highest number of residues located in the favorable part of the Ramachandran plot, as determined by the MolProbity server. The constructed vaccine showed good population coverage when queried on the IEDB population coverage tool. An examination of the ability of the proposed vaccines to bind to the TLR2 immune cell receptor was carried out with the use of a molecular docking study. Since the activation of immune cells is necessary to produce adaptive immunological responses, TLR receptors play a crucial role in innate immunity. It has been demonstrated that TLR2 is responsible for mediating innate immunity against the vaccinia virus [55], which is likewise a member of the orthopoxvirus family to which MPXV also belongs. The results of the molecular docking study showed that the vaccine construct had substantial binding affinities with the active region of the receptor protein. This defines whether or not the vaccine that was designed may produce immunogenic responses that are long-lasting. Studies of molecular dynamics and simulation (MD) were carried out in order to ascertain the degree of stability possessed by the docked complex comprising TLR2 and the vaccine design. Following an examination of the root mean square deviation (RMSD), root mean square fluctuation (RMSF), radius of gyration (Rg), and a number of hydrogen bonds produced by a simulation lasting 100 ns, a stable conformation was observed. MMGBSA analysis of the docked complex provided further evidence of the stability of the system. Validation of a candidate vaccine begins with testing for immunoreactivity using serological evaluation. This is one of the initial phases in the process [56]. It is necessary to express the recombinant protein in an appropriate host in order to do this. Expression systems based on *E. coli* are suitable for the production of recombinant proteins [57,58]. In order to obtain a high degree of expression of our recombinant vaccine protein in *E. coli* K12, codon optimization was carried out prior. There was potential for high-level protein expression in bacteria based on the codon adaptability index of 0.98 as well as the GC content of 50.72 percent. Results from the immunological simulation were found to be compatible with reactions often seen in the immune system. After being repeatedly exposed to the antigen, there was a significant rise in the number of immune responses overall. It was very obvious that memory B-cells had been generated. Additionally, memory T-cells and helper T-cells were also produced. After the first injection, there was a significant rise in the levels of IL-2.

## 5. Conclusions

The monkeypox virus is an emerging and extremely worrying pathogen. A multi-epitope vaccine has been designed using immunoinformatics strategies, with full awareness of the benefits of a peptide vaccine. For the vaccine to be most successful, the incorporation of both T-cell and B-cell epitopes generated from the MPXVgp181 protein were included in the vaccine construct. There is optimism that our vaccination will elicit immunological reactions (both cell-mediated and humoral). Stable and sustained binding potential and interaction between vaccine protein and TLR2 receptor were observed. During the immunological simulation, effective immune responses were seen. However, further research, both in vitro and in vivo, is required to determine whether or not it has the ability to successfully combat the monkeypox virus. The designed protein sequence of the vaccine can be synthesized for conducting expression studies. Once the expression studies are validated, isolated, and purified the vaccine construct can be used for preclinical and clinical studies.

## Figures and Tables

**Table 1:** B-cell linear epitopes.

No.	Antigenic Score	Allergenicity	Start	End	Peptide	Length
**8**	0.5796	Non-allergenic	175	187	LSNGGLPAYYRNG	13
**7**	1.9569	Non-allergenic	163	169	KNTKTGE	7
**3**	0.6951	Non-allergenic	76	84	PDGLDIPLT	9
**2**	1.0008	Non-allergenic	48	64	DQKDYTVTSQFNNYTIG	17

**Table 2:** MHC Class-I epitopes.

Allele	Antigenic Score	Allergenicity	Start	End	Length	Epitope	IC50	Rank
HLA-A*11:01	1.5522	Non-Allergenic	155	163	9	AVYVTMTYK	6.92	0.02
HLA-A*03:01			155	163	9		8.17	0.02
HLA-A*30:01			155	163	9		11.06	0.06
HLA-A*31:01			155	163	9		20.36	0.2
HLA-A*68:01			155	163	9		32.92	0.29
HLA-A*02:03	0.7037		129	138	10	FLTVRKMTRV	6.46	0.08
HLA-A*68:02			129	138	10		98.04	0.58
HLA-A*32:01	0.6748		86	94	9	ITYWSRFTI	22.97	0.05
HLA-A*68:02			86	94	9		43.34	0.34
HLA-B*58:01			86	94	9		43.96	0.25
HLA-A*31:01	0.9515		83	91	9	LTNITYWSR	11.07	0.1
HLA-A*33:01			83	91	9		26.5	0.09
HLA-A*68:01			83	91	9		32.56	0.29
HLA-A*11:01	1.7573		158	166	9	VTMTYKNTK	14.08	0.05
HLA-A*68:01			158	166	9		63.65	0.54
HLA-A*02:03	0.6445		9	17	9	YLIFVTSSI	2.68	0.02
HLA-A*02:01			9	17	9		7.95	0.06
HLA-A*02:06			9	17	9		40.49	0.43
HLA-A*68:02			9	17	9		99.05	0.59
HLA-A*68:01	1.5395		157	166	10	YVTMTYKNTK	34.55	0.3
HLA-A*11:01			157	166	10		51.25	0.31

**Table 3:** MHC class-II epitopes.

Allele	Antigenic Score	Allergenicity	Start	End	Length	Core Peptide	Peptide	Ic50	Rank
HLA-DRB1*15:01	0.6748	Non-allergenic	84	98	15	ITYWSRFTI	TNITYWSRFTIGRAL	26.9	1.5
HLA-DRB1*15:01			83	97	15		LTNITYWSRFTIGRA	28.2	1.6
HLA-DRB1*15:01			82	96	15		PLTNITYWSRFTIGR	29.3	1.8
HLA-DRB1*15:01			81	95	15		IPLTNITYWSRFTIG	39.9	2.6
HLA-DRB1*15:01			85	99	15		NITYWSRFTIGRALF	45.4	3.1
HLA-DRB1*15:01			80	94	15		DIPLTNITYWSRFTI	51.1	3.6
HLA-DPA1*01:03/DPB1*02:01			80	94	15		DIPLTNITYWSRFTI	87	3.7
HLA-DRB1*01:01	1.6046	Non-allergenic	107	121	15	KMSILGVSI	FQKKMSILGVSIECK	83.9	23
HLA-DRB4*01:01			105	119	15		DIFQKKMSILGVSIE	84.2	4.8
HLA-DRB4*01:01			108	122	15		QKKMSILGVSIECKK	89.2	5.2
HLA-DRB4*01:01			106	120	15		IFQKKMSILGVSIEC	90.9	5.3
HLA-DRB4*01:01			107	121	15		FQKKMSILGVSIECK	96.9	5.7
HLA-DRB1*01:01			108	122	15		QKKMSILGVSIECKK	97.8	25
HLA-DRB1*01:01	0.6277	Non-allergenic	7	21	15	LIFVTSSIA	IAYLIFVTSSIASPL	33.7	12
HLA-DRB1*01:01			6	20	15		LIAYLIFVTSSIASP	60.1	19
HLA-DRB1*07:01			6	20	15		LIAYLIFVTSSIASP	61.6	7.2
HLA-DRB1*07:01			4	18	15		YGLIAYLIFVTSSIA	62.1	7.3
HLA-DRB1*07:01			5	19	15		GLIAYLIFVTSSIAS	62.1	7.3
HLA-DRB1*01:01			5	19	15		GLIAYLIFVTSSIAS	70.9	21
HLA-DRB1*15:01			8	22	15		AYLIFVTSSIASPLY	95.8	6.9
HLA-DRB1*01:01			4	18	15		YGLIAYLIFVTSSIA	97.9	25
HLA-DRB1*15:01	0.621	Non-allergenic	122	136	15	LLTFLTVRK	KPSTLLTFLTVRKMT	33.7	2.1
HLA-DRB1*15:01			121	135	15		KKPSTLLTFLTVRKM	34.7	2.2
HLA-DRB1*15:01			123	137	15		PSTLLTFLTVRKMTR	35.6	2.2
HLA-DRB1*15:01			124	138	15		STLLTFLTVRKMTRV	44.4	3
HLA-DPA1*03:01/DPB1*04:02			124	138	15		STLLTFLTVRKMTRV	46.9	1.6
HLA-DPA1*03:01/DPB1*04:02			123	137	15		PSTLLTFLTVRKMTR	49.5	1.7
HLA-DPA1*02:01/DPB1*05:01			125	139	15		TLLTFLTVRKMTRVF	52.3	0.2
HLA-DPA1*03:01/DPB1*04:02			122	136	15		KPSTLLTFLTVRKMT	53.4	1.9
HLA-DRB1*15:01			125	139	15		TLLTFLTVRKMTRVF	53.9	3.8
HLA-DPA1*02:01/DPB1*05:01			124	138	15		STLLTFLTVRKMTRV	55.3	0.22
HLA-DPA1*02:01/DPB1*05:01			126	140	15		LLTFLTVRKMTRVFN	60.7	0.25
HLA-DPA1*02:01/DPB1*05:01			123	137	15		PSTLLTFLTVRKMTR	65.3	0.29
HLA-DPA1*02:01/DPB1*05:01			121	135	15		KKPSTLLTFLTVRKM	80.7	0.45
HLA-DPA1*02:01/DPB1*05:01			122	136	15		KPSTLLTFLTVRKMT	95.3	0.66
HLA-DRB5*01:01			122	136	15		KPSTLLTFLTVRKMT	96.7	12
HLA-DPA1*02:01/DPB1*05:01			120	134	15		CKKPSTLLTFLTVRK	98.5	0.68
HLA-DRB1*01:01	1.0246	Non-allergenic	124	138	15	LTFLTVRKM	STLLTFLTVRKMTRV	23.5	7.9
HLA-DRB1*01:01			123	137	15		PSTLLTFLTVRKMTR	27.4	9.3
HLA-DRB1*01:01			125	139	15		TLLTFLTVRKMTRVF	29.1	9.8
HLA-DRB1*01:01			122	136	15		KPSTLLTFLTVRKMT	34.3	12
HLA-DRB1*01:01			126	140	15		LLTFLTVRKMTRVFN	42.9	14
HLA-DRB1*01:01			121	135	15		KKPSTLLTFLTVRKM	49	16
HLA-DRB1*01:01			127	141	15		LTFLTVRKMTRVFNR	81.8	23
HLA-DPA1*03:01/DPB1*04:02	0.6804	Non-allergenic	31	45	15	SFNSVEVLV	EDKSFNSVEVLVSLF	60.5	2.3
HLA-DPA1*03:01/DPB1*04:02			32	46	15		DKSFNSVEVLVSLFP	60.6	2.3
HLA-DPA1*03:01/DPB1*04:02			33	47	15		KSFNSVEVLVSLFPD	68.3	2.8
HLA-DPA1*03:01/DPB1*04:02			30	44	15		SEDKSFNSVEVLVSL	69.3	2.9
HLA-DPA1*02:01/DPB1*01:01			33	47	15		KSFNSVEVLVSLFPD	95.4	1.7
HLA-DRB1*01:01	0.6068	Non-allergenic	19	33	15	YIPVIPPIS	SPLYIPVIPPISEDK	15.3	4.9
HLA-DRB1*01:01			18	32	15		ASPLYIPVIPPISED	20.2	6.7
HLA-DRB1*01:01			20	34	15		PLYIPVIPPISEDKS	20.5	6.8
HLA-DRB1*01:01			17	31	15		IASPLYIPVIPPISE	22.2	7.5
HLA-DRB1*01:01			21	35	15		LYIPVIPPISEDKSF	33	12
HLA-DRB1*01:01			16	30	15		SIASPLYIPVIPPIS	45.2	15
HLA-DRB1*09:01			17	31	15		IASPLYIPVIPPISE	81.6	8.1
HLA-DRB1*09:01			18	32	15		ASPLYIPVIPPISED	83.2	8.2
HLA-DRB1*04:01	1.6018	Non-allergenic	19	33	15	YVTMTYKNT	SPLYIPVIPPISEDK	91.7	3.8
HLA-DRB1*09:01			19	33	15		SPLYIPVIPPISEDK	95.5	9.6
HLA-DRB1*04:05			18	32	15		ASPLYIPVIPPISED	96.2	5
HLA-DRB1*04:05			19	33	15		SPLYIPVIPPISEDK	96.2	5

**Table 4:** Hydrogen bonds between Chain A (TLR2) and Chain B (vaccine) of TLR2—vaccine complex.

Sl. No	Atom No.	Residue Name	Residue No.	Chain	Hydrogen Bond	Atom No.	Residue Name	Residue No.	Chain
1	53	A.R.G.	32	A	<-->	5666	GLU	33	B
2	56	A.R.G.	32	A	<-->	5665	GLU	33	B
3	100	LYS	37	A	<-->	5624	GLU	29	B
4	541	SER	85	A	<-->	7215	ARG	207	B
5	747	SER	108	A	<-->	7382	ARG	221	B
6	1211	ARG	155	A	<-->	7328	THR	217	B
7	1214	ARG	155	A	<-->	7331	THR	217	B
8	1214	ARG	155	A	<-->	7361	TRP	219	B
9	1243	MET	159	A	<-->	7171	TYR	202	B
10	1442	GLU	178	A	<-->	7346	TRP	219	B
11	2742	THR	313	A	<-->	5397	THR	6	B
12	3044	ARG	340	A	<-->	5406	ASP	7	B
13	3590	GLN	396	A	<-->	7480	ILE	231	B
14	3836	LYS	422	A	<-->	7524	SER	235	B
15	4435	TYR	483	A	<-->	5485	GLU	15	B
16	4641	LYS	505	A	<-->	5484	GLU	15	B

**Table 5:** Salt bridges between Chain A (TLR2) and Chain B (Vaccine) of TLR2–vaccine complex.

Sl. No	Atom No.	Residue Name	Residue No.	Chain	Salt Bridge	Atom No.	Residue Name	Residue No.	Chain
1	53	A.R.G.	32	A	<-->	5665	GLU	33	B
2	100	LYS	37	A	<-->	5624	GLU	29	B
3	3044	ARG	340	A	<-->	5406	ASP	7	B
4	4641	LYS	505	A	<-->	5484	GLU	15	B
5	5220	LYS	561	A	<-->	5666	GLU	33	B

**Table 6:** Formation of hydrogen bonds and salt bridges and their number with respective amino acid residues between protein and ligand–protein.

Residue	Closest	Distance	Specific Interactions	H.B.	Salt Bridges
A:32:Arg	B:40:Val B:41:Ala B:36:Ala B:39:Pro	3.1 A 3.3 A 3.7 A 3.7 A	1× hb to B:40:Val 1× hb to B:41:Ala	2	0
A:58:Asp	B:14:Lys	2.6 A	1× hb, 1× salt bridge, to B:14:Lys	1	1
A:344:Glu	B:221:Arg	3.0 A	1× hb to B:221:Arg	1	0
A:390:Gln	B:195:Thr B:194:Val	2.6 A 3.2 A	1× hb to B:195:Thr	1	0
A:460:Glu	B:199:Lys	2.7 A	1× hb to B:199:Lys	1	0
B:14:Lys	A:58:Asp	2.6 A	1× hb, 1× salt bridge, to A:58:Asp	1	1
B:40:Val	A:32:Arg A:33:Asn A:53:Ala A:77:Asn	3.1 A 3.5 A 3.6 A 3.7 A	1× hb to A:32:Arg	1	0
B:41:Ala	A:32:Arg A:33:Asn	3.3 A 3.5 A	1× hb to A:32:Arg	1	0
B:195:Thr	A:390:Gln A:416:Thr	2.6 A 3.5 A	1× hb to A:390:Gln	1	0
B:199:Lys	A:460:Glu A:440:Tyr	2.7 A 3.1 A	1× hb to A:460:Glu	1	0
B:221:Arg	A:344:Glu A:315:Arg	3.0 A 3.8 A	1× hb to A:344:Glu	1	0

hb = hydrogen bond, A = protein chain, B = ligand–protein chain.

## Data Availability

Not applicable.
